# Supervision of redeployed surgical trainees during the COVID-19 pandemic: what have we learnt and how can we improve?

**DOI:** 10.1186/s12909-023-04275-4

**Published:** 2023-05-09

**Authors:** Esther V Wright, Nicholas D Haden, Kirsten Dalrymple

**Affiliations:** 1 Department of Surgery and Cancer, Imperial College London, South Kensington Campus, SW7 2AZ London, UK; 2 Royal National Orthopaedic Hospital, Stanmore, HA7 4LP UK

**Keywords:** COVID-19, Surgeons, Critical care, Education, Patient safety

## Abstract

**Objective:**

When cases of patients presenting with Coronavirus Disease 2019 (COVID-19) overwhelmed existing services in the United Kingdom (UK), surgical trainees were redeployed to assist frontline efforts. This project characterises the effects of redeployment on the supervision of these trainees. The resulting generation of practical recommendations could be implemented for future crises.

**Design:**

A qualitative approach was utilised, comprised of seven phenomenological interviews with surgical and intensive care consultants, as well as redeployed surgical trainees. Interview recordings were transcribed and subsequently analysed using Thematic Analysis.

**Setting:**

The project utilised participants currently in surgical training within the London deanery across a variety of surgical specialties representing several UK National Health Service (NHS) Trusts.

**Participants:**

Three types of participants were interviewed. Four interviews were conducted with redeployed surgical trainees, across all stages of training, in full time employment who were redeployed for two weeks or more. One interview was conducted with an educational supervisor of multiple redeployed trainees. The third group comprised two consultant intensivists who supervised redeployed trainees within their respective departments.

**Results:**

Four themes were developed: ‘Responding to an unforeseen crisis’, ‘Maintaining surgical identity and culture; A fish out of water?’, ‘Trainee supervision and support’ and ‘Preparation and sequelae’. Participants described a sense of obligation to the pandemic effort. Many described a significant interruption to training, however communication of this to surgical supervisors was suboptimal with minimal mitigation. Supervisors on the frontline were challenged by the assessment of trainee competence and acceptance into a new community of practice. Both trainees and supervisors described the management of uncertainty, advocating for the use of reflective practice to ensure preparation for the future.

**Conclusion:**

This project presents an insight into several potentially long-lasting effects on surgical training. The recommendations generated may be applicable to trainees returning to work from time out of training, increasing the utility of this work.

## Background

### Supervision in surgical training

The process of training a surgeon is complex, balancing the development of individual expertise with the requirements for patient safety and service provision. Ensuring that trainees are adequately supervised throughout this journey has been demonstrated not only to improve patient outcomes but also trainee satisfaction and rate of skill acquisition [1, 2]. Proctor outlined three roles of an effective supervisor: normative (administrative), formative (educational), and restorative (supportive) [3]. When translated into clinical practice, these roles encompass several fundamental components of training including providing feedback, education planning and regulation of appropriate clinical duties.

Within UK surgical training, the General Medical Council (GMC) regulates the level of supervision that surgical trainees in the UK should expect [4]. Trainees are supervised by a minimum of two individuals: one educational supervisor (ES) who oversees longitudinal development, and either one or multiple clinical supervisors (CS) specific to their rotation who supervise day to day clinical activity. ES must be allocated protected time for these additional duties, should be appropriately trained and provided with necessary support [5].

Despite formalisation of trainee supervision, this structure alone has not been sufficient to manage variability in training opportunities and experience. More recently, the coronavirus disease 2019 (COVID-19) pandemic has resulted in a significant and unprecedented challenges to both provision of surgical services and education, with direct effects on face-to-face training and provision of effective supervision.

### The healthcare response to the COVID-19 pandemic

In response to exponential increases in COVID-19 cases, restructuring of National Health Service (NHS) facilities occurred both rapidly and on mass scale, resulting in significant change to standard service provision. Subsequently, surgical trainees were redeployed to areas of clinical priority. These included Accident and Emergency (A&E), Intensive Care Unit (ICU) and acute medical or COVID-19 specific wards, a phenomenon experienced internationally [6].

A small volume of research has been carried out to investigate the effects of redeployment on trainees. Juan et al. conducted a systematic review of papers concerning the redeployment of a wide spectrum of healthcare workers including nursing staff and supporting members of the multidisciplinary team (MDT). The review suggested that key features of successful redeployment included identifying and utilising trainee skillsets, developing flexible strategies and preparation including staff induction and sustained training [7]. Payne et al. also focused on the redeployment of surgical trainees to ICU. Their questionnaire, distributed to over 90 participants, highlighted a number of concerns for trainee wellbeing such as personal and family health concerns, isolation, lack of operative experience and future career uncertainty [8]. Both papers provided a cross sectional representation of the immediate effects of redeployment on trainee workload, experience and education. They recommended the need for future research, exploring redeployment with a wider focus and appreciating the broader effects on trainee welfare as opposed to missed surgical experiences alone. When considered with regards to Proctor’s model, little emphasis was placed on the formative and restorative elements, which address the role of the supervisor with regards to knowledge and skill development as well as emotional and psychological support respectively [3]. This current project expands on this existing research, utilising a phenomenological approach to qualitative methodology with the purpose of developing further understanding of how surgical trainees were affected by redeployment professionally and personally and establishing whether this process could be improved.

## Method

As this research question focuses primarily on the experiences of individuals, it lends itself to a qualitative methodology, including principles from Husserl’s phenomenology. Husserl developed phenomenology as a means to conceptualise the lived experiences of people and their deeper meaning [9]. We hoped that evaluating and reflecting upon the real-life experiences of trainees, and their supervisors, during this period would facilitate an understanding of the supervision requirements, and potential deficits, that would not be possible utilising alternative research methodologies.

### Ethical approval

This research was carried out in accordance with the declaration of Helsinki and was granted ethical approval by the Imperial College Education Ethics Review Process (Study Number EERP2021- 067). All participants provided informed consent and were required to complete and return a signed consent form prior to participation.

### Interview participants

Interview participants were selected via purposive sampling. Recruitment occurred primarily from core surgical trainees (CST) within the London deanery, as a higher proportion of trainees inside central London had been redeployed compared with national averages. Awareness of the project was generated through digital advertisements distributed via social media platforms and posters placed in multiple London teaching hospitals. ES, both surgical and anaesthetic/ICU were recruited in a similar fashion. Snowballing methods were utilised to reach additional participants, who were contacted directly, and all agreed to participate [10].

### Eligibility criteria

Three separate groups of participants were recruited, all with specific eligibility criteria as demonstrated in the Table 1. Only trainees currently enrolled in a national training programme were included, as availability of training opportunities for those outside of a training programme is highly variable. CS were excluded from this study as their role generally is to oversee clinical practice within specific short-term placements.

**Table 1:** Inclusion and exclusion criteria for survey and interview participants

Group	Inclusion	Exclusion
1. Redeployed surgical Trainees	Surgical trainee (CT1/ST1 to ST8) in full time training Any surgical specialty Redeployed for > 2 weeks Redeployed to A&E, ICU, Acute medicine or COVID specific ward	Not in nationally accredited training programme Part-time redeployment
2. Educational Supervisors of redeployed surgical trainees	ES of redeployed surgical trainee ES appointed by Trust (Formal role) Redeployed trainee in question must fulfil the criteria for inclusion	CS role only
3. Educational supervisors receiving redeployed surgical trainees to their departments	ES receiving redeployed trainee into your department ES appointed by Trust (Formal role) Redeployed trainee in question must fulfil the criteria for inclusion	CS role only

### Interviews

Seven one-to-one remote semi structured interviews (SSI) were conducted via the videoconferencing platform, Zoom, totalling 227 min. Two SSIs were conducted with ICU consultants, one with a consultant plastic surgeon and four with core surgical trainees representing Ear, Nose and Throat surgery (ENT), Trauma and Orthopaedics (T&O) and Plastic surgery. The interview setup, remote individual interviews, was designed to encourage individuals to relive their experiences without the external influence of others’ opinions within the wider social context. SSIs were chosen to establish continuity within question delivery across interviews [11]. In addition, follow up questions were pre-established to allow the interviewer to delve further into specific areas of interest raised. Thematic Analysis (TA) was employed as a framework for analysing the resulting interview data using Braun and Clarke’s six-phase process [12]. Saturation was determined at stage four of this process (reviewing themes), where no further themes emerged from the data.

### Data handling

Interviews were transcribed using the software Otter.ai. Interview recordings were then reviewed alongside the transcription and corrections made prior to data analysis. The corrected transcripts were uploaded to the programme NVivo (Release 1.0), a qualitative data analysis software. The software was used to generate data codes and identify sub-themes and themes.

Interview participants were coded according to Table 2 below to ensure pseudo anonymity at time of data transcription and analysis:

**Table 2:** Interview participants coded by professional role

Code	Role
ES1	ES - Plastic surgery
T1	CST – Plastics
T2	CST – Plastics
T3	CST – ENT
T4	CST – T&O
RES1	ICU/ anaesthetic ES
RES2	ICU/ anaesthetic ES

## Results

The remote interviews resulted in the development of 11 sub-themes from which four themes were generated. These are as follows:

1. Responding to an unforeseen crisis.
  - Change in workplace environment
  - Prioritisation
  - Uncertainty
2. Maintaining surgical identity and culture: A fish out of water?
  - Acceptance into a new community of practice.
  - Managing emotions and attitudes.
3. Trainee supervision and support.
  - Impaired Communication
  - Trainee wellbeing
  - Establishing competence
4. Preparation and sequelae.
  - Identifying missed opportunities and mitigating losses
  - Innovation and change in practice
  - Career planning

### Theme one: responding to an unforeseen crisis

- Change in workplace environment

Throughout the interview process, both trainees and consultants referenced the concept of responding to a crisis, including their willingness to adapt and contribute to the unprecedented circumstances brought about by the pandemic.

Participants spoke about “*the crazy time”* (T4) and “*the magnitude of the pandemic”* (T2), stating that “*everyone was overwhelmed”* (RES2). RES 2, an ITU consultant, compared her department to a warzone:

“*Sheets hanging everywhere, it was looking like a war….no circulation, everyone was dripping sweat” (*RES2)

1. Prioritisation

Regardless of their position or personal experience, trainees unanimously agreed that redeployment was necessary and suggested that their own training needs were no longer the priority during this time. Examples of these statements included:

“*When you put the shoe on the other foot, we have to realise that redeployment was something that was a necessity in some regard because we required feet on the ground”* (T2)

“*I think during the crisis, that during a pandemic, you have to reorder your thinking of your own priorities”* (T2).

This mindset shifted in some trainees however as the pandemic wore on and entered a second wave.

“*I understand that this is all in the context of a pandemic, it’s all new ground. However, weeks and weeks and weeks have gone by, and we haven’t contributed anything towards the pandemic during our redeployment…. we weren’t particularly needed just a lot of waiting around”* (T1).

 

1. Uncertainty

The concept of uncertainty was raised on multiple occasions in relation to surgical trainee redeployment both by trainees and ES.

*The thing is we didn’t know how long it would be, for instance to be a broad impact on my training. But you know say okay well maybe it’s going to be two or three months, let’s see how it goes” (*T3).

One ICU consultant highlighted the effect this uncertainty had on their judgement regarding appropriate levels of supervision, and the effect this may have on the trainees themselves.

*“Your own concerns about leaving trainees you don’t really know that well unsupervised... ‘hopefully this didn’t happen too much on our unit but at the least it’s probably anxiety generating for trainees“*(RES1).

### Theme two: maintaining surgical identity and culture: a fish out of water?

-  Acceptance into a new community of practice

The notion of working alongside colleagues, from a variety of different communities of practice, is a challenge raised by redeployment. Some of the interviewees highlighted discrete differences between surgical trainees and their anaesthetic/ICU colleagues both clinically and attitudinally.

*“We were kind of dropped in this medical ward where no one wanted us to be there…we just chilled and the medics didn’t want us to do anything, we weren’t going to do anything, so we were just hanging out as a group of surgeons”* (T1).

T4 described a more positive experience towards the end of redeployment and alluded to the potential for long-term improvement in her interprofessional working practices.

*“I did actually get some ITU experience and made good working relationships with more of the anaesthetic members of the department… so when the sort of quarantine started lifting, it was even better working relationships all around in theatre, which you know facilitates speedier cases and things like that”* (T4).

An inability to understand the role of ICU doctors and their clinical demands left surgical trainees feeling disconnected at times from their existing ES. T3, a surgical trainee who was redeployed to ICU for over eight months, displayed insight into her own supervisor’s difficulty to understand her new role.

“*It would have been nice to have a bit more involvement if that understanding was there but it’s very difficult, I think, for a surgical consultant”* (T3)

2b. Managing emotions and attitudes

Multiple trainees described negative emotions towards their redeployment, with a reluctance to make their attitudes public.

*“No trainee in their hearts of hearts would be completely content to be redeployed because there is an impact on their training and there is an impact on a personal level” *(T2).

ES1 a plastic surgery consultant reported his own trainees making similar comments regarding their aversion to redeployment. The way in which they communicated this to him avoided making a direct complaint, despite this, he reports understanding the latent meaning in their comments. He then went on to suggest that surgeons took pride in their involvement with the pandemic.

“I think we knew it was just for a small period and actually there was an opportunity for surgeons to show that they work harder than they need …we normally would be the ones who have volunteered the greatest number of trainees and as consultants we would be the last there and the first ones to get changed, so kind of showing them actually this is what we are like every day” (ES1)

### Theme three: trainee supervision and support

- Impaired Communication

ES and trainees highlighted the poor communication they experienced throughout redeployment. Phrases such as “*third hand”*, “*lack of transparency”* and “*word of mouth”* were used by trainees to describe the way in which they were informed that they would be redeployed. When asked whether his role as an ES had changed during the pandemic, ES1 responded with “*I think it changed completely because I didn’t see my trainees”* reinforcing the idea of communication breakdown.

Poor communication with supervisors may be explained by the fact that supervisors themselves had disruption to their clinical practice. ICU/anaesthetic supervisors were busy managing their units and supervising trainees and some surgical consultants found themselves redeployed or with altered clinical responsibilities. T1 acknowledged the effects on her ES availability.

“*My educational supervisor was also redeployed, and the majority of his lists were cancelled, and he was also on the proning team and doing things…I think he was suffering as well. The impression I got was that obviously it wasn’t ideal for him either”* (T1).

3b. Trainee wellbeing

Participants reported limited or ineffective initiatives for supporting the wellbeing of staff, despite the exceptional circumstances and level of distress they experienced, though this improved over the course of the pandemic. RES2 described being directed to an app for wellbeing support, which she stated was not particularly useful and appeared to be a method of appeasement. She also highlighted the lack of debriefing, particularly in the first wave as an area that could be targeted for improvement.

“A *lot of people have seen bad things, young people die and a lot of families, kids crying for their dead parents, and things like this, and there has been no official debriefing for this”*(RES2).

T3 suggested that there was more of a focus on wellbeing in the second wave and that her ES would have been able and willing to support her psychologically.

“*Looking at individual circumstances at home and if people are shielding and living with vulnerable people, I think that’s important to consider and I think that was done a bit better in the second wave “*(T3)

3c. Establishing competence

Multiple trainees recognised the unique learning opportunity afforded by being redeployed to ICU and described a specific desire contribute to and establish competence in a new discipline. T1 highlighted this when she stated,

“*I wouldn’t have minded going to ICU and learning how to use the ventilators and learning how to deal with more critically unwell patients”.*

On the other hand, T4 described “*doing very boring, mundane jobs like discharge letters and scribing on ward rounds”* when placed on ITU, as she felt that learning opportunities were seldom afforded to surgical trainees. She then explained her reasoning behind why this occurred, stating

“*That was probably the job of the ICU supervisor consultants, on that day, to make sure that everyone was working within their remit and that it was safe”.*

### Theme four: Preparation and sequalae

- Identifying missed opportunities and mitigating losses

Trainees repeatedly emphasised missed learning opportunities throughout their redeployment. These occurred in both their home speciality and when on ICU or medical wards. T1 described a paradox, in that learning opportunities within her home specialty increased during the pandemic, however her ability to utilise them was diminished.

*“A lot of our service was continuing as before because the vast majority of our workload is trauma … the amount of procedures that we were doing in clinic was increasing. Not only did I feel like I was missing out, but actually it was quite a critical period where I felt like I could have done a lot more procedures than I would usually have done”* (T1).

T2 understood that operating experience, despite being his priority would be difficult to obtain and suggested some potential methods of mitigating for this lost experience.

*“The main problem would be about operating time and surgical exposure… if you don’t have the surgical patients in the hospital, it’s almost impossible unless we were to look at avenues such as virtual reality. Basically, technological adjuncts, which would have to be well in place in the training process”* (T2)

4b. Innovation and change in practice

The pandemic also brought about significant changes in practice. Within many surgical specialties increasing numbers of cases were either managed non-operatively or a general anaesthetic was avoided unless essential. ES1 discussed the occurrence of this phenomenon within plastic surgery and suggested that not only did this provide him the opportunity to supervise and teach trainees new methods of operating, but that it has changed his practice going forwards.

“*It meant that we did a lot more things under local, so that was a good training opportunity, to teach people how to do flexor tendons and fractures under local anaesthetic” (*ES1)

 

4c. Career planning

T3 explained that in her experience, guidance with respect to career progression often come from interaction with her supervisors. Both T2 and T4 then went on to explain their own concerns regarding career progression and the effects of redeployment on their success at application for an ST3 post.

“*COVID changed the whole ST3 application and resulted in not getting a job, which is a devastating blow, and, and then obviously not being in surgery for a few months I was worried that that was then going to impact my skill set and ability to get an ST3 job in the next recruitment process as well”* (T4)

## Discussion

Analysis demonstrates that the redeployment during the COVID-19 pandemic did have a direct effect on the lives of surgical trainees and their supervisors, both within the workplace and on a wider scale, with demonstrable effects on wellbeing. Both trainees and their supervisors did suggest possible strategies for improving this process, some generalisable such as improvement in communication, and others more specific, for example identifying and mitigating for missed training opportunities. Positive effects were not overlooked however, with improved departmental relations highlighted repeatedly, alongside opportunities for new skill acquisition.

Within theme one, participants alluded to a both a sense of chaos and unfamiliar working conditions. The ability of professionals to respond to a crisis and modify their practice relates to the theory surrounding routine and adaptive expertise. The routine expert achieves mastery in their field, within the realms of acceptable existing practice, whereas the adaptive expert utilises this knowledge to innovate [13]. In unforeseen circumstances such as a pandemic, those with the ability to innovate and adapt may take changes to their working environment and scope of practice in their stride, perhaps even seeing this as an opportunity to drive innovation further.

Ideas raised within theme two depict the social nature of learning and the way in which redeployed surgical trainees responded to their new working environments. Despite understanding the necessity for redeployment, both trainees and the surgical ES interviewed, described at times feeling like outsiders, being under-utilised, and viewed purely as service providers. Wenger’s theory of the Community of Practice, alongside the concept of legitimate peripheral participation, go a long way to describing this sociocultural nature of learning [14, 15]. Within the cohort of redeployed trainees, learning opportunities and supervision were clearly afforded to those who had been “accepted”. Furthermore, those willing to engage with their new community seemed to benefit most, as in the case of T4. Integration into a new community involves learning the appropriate vocabulary as well sharing experiences and observing the practice of senior community members.

In addition, Lave and Wenger raise the concept of brokering knowledge, suggesting that some individuals can introduce new elements of practice between communities. For this to be successful, the new elements must be sufficiently novel to gain the attention of the receiving community, similarly the individual must be considered by them as legitimate [16]. This phenomenon may explain the way in which knowledge was shared during this critical time; the result of forced exposure to a new community of practice, bringing with it the opportunity to observe new practice and share existing knowledge.

During the pandemic, there was a necessity for acceleration of these processes, meaning that those who were more active and involved within the team were afforded opportunities more frequently and therefore may have achieved acceptance at an earlier stage [17]. An understanding of this process depicts a theory-informed solution; ensuring that trainees to be redeployed have a basic grounding and understanding of the principles of their new department. This could be facilitated through a teaching programme directed at trainees to be redeployed, however this would require resources that may not have been available during this original period of crisis.

Concerns regarding ineffective communication were prominent within theme three. Whilst trainee concerns included duration of redeployment, expected duties and the desire to feel included and appreciated, these details were not relayed reliably. The inability to achieve effective communication is arguably implicated in a supervisor’s ability to support their trainees. Surgical ES reported being unaware of the expected duties of their redeployed trainees, similarly some trainees did not consider that their supervisors had the clinical experience of ICU and medicine to assist them successfully. Such issues may have been a source of anxiety for both trainee and supervisor, which, with adequate preparation may be avoidable. This issue could be addressed from two angles, either by educating existing supervisors regarding the likely duties and expectations of their redeployed trainees, and/or by assigning redeployed surgical trainees a new supervisor within their temporary department, to act as a point of contact and clinical support.

Theme four centred around reflective practice. Utilising and reflecting on experiences described during the interviews may assist with directing improvements to the redeployment process. Every trainee interviewed commented on their concern regarding lack of operative exposure, the potential to deskill and associated repercussions on career progression. Suggestions made by trainees and supervisors to combat this included: part-time or needs based redeployment, additional observer-ships, extension of training and use of simulation-based training. It has been repeatedly demonstrated that skills developed in the simulation setting are directly transferrable to the clinical environment [18]. Furthermore, Kneebone describes simulation as a safe and realistic adjunct to existing surgical training, however he accepts its limitations with regards to technological advancement and cost of implementation [19]. Such tools could be considered at either the point of return to specialty, or provided to surgical trainees throughout redeployment, as a method of “touching base”and aiming to prevent deskilling at a minimum.

### Recommendations

Given the results and discussion of this qualitative study we suggest the following initiatives:

1. The requirement for redeployment of surgical trainees should be subject to frequent reassessment to avoid unnecessary relocation and interruption to training.
2. Communication regarding trainee redeployment should be delivered directly to trainees from their supervisors, it should include details surrounding the provisional duration as well as expected duties.
3. ES of redeployed trainees should ensure regular communication throughout the duration of redeployment, this can be face to face or via virtual/telecommunication methods.
4. Redeployed trainees should be assigned a new CS within the department to which they are assigned for the entire duration of their redeployment.
5. Redeployed trainees should be provided with an induction to their new department, where possible trainees should undertake training in the basic required skillset for their new role.
6. Where possible, the skillset and competence of trainees should be established prior to redeployment (via direct communication with the trainee or review of placement records) and relayed to their new CS.
7. On return to specialty, trainees along with their ES, should identify missed learning opportunities and formulate a realistic plan to address these.
8. Surgical departments should consider the provision of virtual teaching sessions/ e-learning for redeployed trainees. Simulation sessions may be considered as an adjunct to prevent de-skilling.
9. Redeployed trainees should have periodic opportunities to debrief with their new and existing ES to and should be provided with appropriate contact information and resources for any wellbeing concerns.
10. On conclusion of redeployment, trainees and supervisors should conduct a formal debrief on their experiences and utilise this to inform future occurrences.

### Limitations

At the time of data collection, I was practising as a core surgical trainee within the London deanery and had myself experienced COVID-19 redeployment. This provided both the motivation to undertake this project, as well as a unique understanding of events described by the participants. I was aware, however, of an unavoidable element of confirmation bias. This may have occurred at three key points, the delivery of questions during interview, data interpretation and framing of results. To mitigate for this, I aimed to ask relatively open questions and followed the pre-conceived follow up questions and prompts during interview, aiming to avoid asking leading questions or offering my own opinion. When reflecting on the interviews undertaken with consultants specifically, I was aware of maintaining the boundaries of our professional relationship, which may have limited the range of questions delivered. I am optimistic that my insight into my own position as the researcher will have helped me to maintain objectivity in data analysis and presentation.

The planning and conduction of this project was unexpectedly interrupted by a surge in COVID-19 cases between January and March 2021. Consequently, the time restrictions on completion of this project were significantly affected as was my ability to recruit other surgical trainees, all of whom were in similar situations. Furthermore, I experienced the greatest difficulty recruiting consultant surgeons, due to the amendments to their working patterns.

From a logistical perspective, most trainees and all the ES interviewed were from London deaneries. These geographical locations were the most disrupted by COVID-19 with the highest caseloads and subsequent redeployment. It is therefore possible that the experience of trainees interviewed was not representative of that nationally, however my position as a London trainee made recruitment outside of this location challenging.

## Conclusion

The effects of a global pandemic on modern surgical training may never be experienced or studied again within our lifetimes, representing a unique opportunity to gain insight into this exceptional occurrence. Intending to reflect on and hoping to improve what had been a challenging time professionally, personally, and psychologically for surgical trainees has also yielded a series of recommendations with potential widespread utility for the future. Existing research into the field of supervision in redeployment comes largely from opinion pieces and survey-based data, meaning that this project is one of the first to utilise qualitative methodology, in part, as a voice for surgical trainees and their supervisors.

Participants discussed a plethora of pertinent topics, for example, development of surgical identity, managing uncertainty, and establishing competence, all of which have been widely described in educational literature. It is evident that although the pandemic brought novel and unique challenges to the world of surgical education, its arrival also magnified unresolved inefficiencies and pitfalls affecting the supervision of surgical trainees. Whilst offering a glimpse into the experiences and interruption to training of the participants interviewed, this project represents only a snapshot of a wider problem and tells us little of the possible long-term repercussions. The potential for a follow up study would provide further clarity on whether these trainees faced continued challenges to their training following this disruption.

## Data Availability

The datasets used and/or analysed during the current study are available from the corresponding author on reasonable request.

## Abbreviations

COVID-19: Coronavirus Disease 2019
UK: United Kingdom
NHS: National Health Service
GMC: General Medical Council
ES: Educational Supervisor
CS: Clinical Supervisor
A&E: Accident and Emergency
ICU: Intensive Care Unit
MDT: Multidisciplinary Team
CST: Core surgical trainee
SSI: Semi Structured Interview
T&O: Trauma and Orthopaedics
ENT: Ear, Nose and Throat
TA: Thematic Analysis
